# Substance-use disorder in high-functioning autism: clinical and neurocognitive insights from two case reports

**DOI:** 10.1186/s12888-015-0541-7

**Published:** 2015-07-07

**Authors:** Laurence Lalanne, Luisa Weiner, Benoit Trojak, Fabrice Berna, Gilles Bertschy

**Affiliations:** 1 Department of Psychiatry, Strasbourg University Hospital, 1 place de l’Hôpital, 67000 Strasbourg, France; 2 INSERM 1114, FMTS, Strasbourg University Hospital, 1 place de l’Hôpital, 67000 Strasbourg, France; 3 Department of Psychiatry and Addictology, Dijon University Hospital, Dijon, France; 4 EA 4452, LPPM, University of Burgundy, Dijon, France

**Keywords:** Substance-use disorders, Autism, Cognitive disabilities

## Abstract

**Background:**

Low prevalence of substance-use disorder has been reported in adults with autism. However, on a superficial level, adults with high-functioning autism (HFA) display a ‘normal’ façade when they drink alcohol, which may explain why their alcohol dependency is not better diagnosed.

**Case presentation:**

Here, we report two cases of HFA adults who use alcohol and psychostimulants to cope with their anxiety and improve their cognitive abilities and social skills. We analyze how neurocognitive traits associated with HFA may be potential triggers for substance-use disorder.

**Conclusion:**

Better identification of autism and its cognitive impairments, which may be vulnerability traits for developing substance-use disorders, could help improve the diagnosis and treatment of substance-use disorders among this population.

## Background

Several psychiatric co-morbidities have been associated with High-Functioning Autism (HFA), but a surprisingly low prevalence of substance-use disorders (SUDs) has been reported in this population [1] (results of a study involving 122 patients). Furthermore, another study [2] has reported that teenagers with HFA had a relatively low risk of developing a co-morbid SUD. This is attributed to the fact that they display few sensation-seeking traits and an introverted personality overall. Sizoo *et al.* [3] showed higher rates of co-morbid SUD in patients suffering from Attention deficit hyperactivity disorder (ADHD) compared with those with Autism Spectrum Disorder (ASD) (58 % versus 30 %), but their study did not focus on HFA. The aforementioned studies focused on the prevalence of SUDs among individuals with ASD, rather than on the prevalence of patients suffering from ASD among patients with SUDs. In his book, Matthew Tinsley, an adult with HFA and alcohol dependency, reported that alcohol use in this population may be a way for them “to cope with their anxiety, to maintain friendships, to give access to a whole host of relationships and even to sustain careers” [4]. According to these authors, adults with HFA may superficially display a ‘normal’ façade when they drink alcohol, which may explain why their alcohol dependency is not better diagnosed. On a neuropsychological level, similar to what has been reported in alcohol-dependent patients, the maladaptive use of alcohol, which is probably underestimated in this population, might be connected with their poor social problem-solving skills, such as poor self-reported empathy, and impaired emotion recognition skills [5]. In addition to symptoms of anxiety, which are part of the clinical picture, the main neurocognitive traits associated with autism are social cognition impairments, a local processing bias or weak central coherence, and executive dysfunction [6]. These traits may be risk factors for developing alcohol dependency because they are related to anxiety and social problem-solving skills. To examine this hypothesis, in this paper we present a detailed clinical and neurocognitive description of two patients with HFA and a co-morbid SUD.

## Case presentation

### Mr. B. and Mr. T.

**Mr. B.**, a 45-year-old single man, was admitted to our psychiatric department for the first time in 2009. He was socially withdrawn. Upon being admitted to our department, he requested treatment for anxiety and major depression co-morbid with alcohol dependency. As he described it, his depressive episodes in the past could rapidly switch to mania. The current episode had lasted for 2 months. When we met him, he presented with a sad mood associated with a worsening of symptoms in the morning hours, early-morning waking, psychomotor retardation, weight loss, and excessive guilt. His constant anxiety led him to spend the whole day, and sometimes the whole night, wandering the streets. He had a very rigid schedule and an impressive calendar memory. His social behavior was aloof and naïve. For example, due to his difficulty with mental state attribution, he might give his house keys to complete strangers if asked to do so. His communication style was very detailed, egocentric and literal. He only showed an interest in talking about his main passions, such as detailed historical events and politics. Mr. B. explained that ever since early childhood he had always been lonely and had always lacked “social intelligence.” He displayed many types of ritualistic and restricted behaviors. For example, he bought the same food in the supermarket every day, his time was spent on the same activities, at the same time, every day, and his interests were restricted to history, as well as international and national politics. When something unexpected happened, like a tram being late, he showed increased anxiety and an intense craving for alcohol and smoking which led him to consume both substances in a compulsive manner. The patient reported a heightening of his attentional capacity after smoking, and lower anxiety levels after drinking alcohol. Owing to alcohol intoxication, he stopped tending to his apartment and accumulated a lot of debts. In the past, he had tried to cut back on his drinking, but such attempts had lasted less than a week. His addiction to alcohol and cigarettes began when he was an adolescent. During the first interview, he was diagnosed as tobacco and alcohol dependent according to DSM-IV criteria (Fagerström score: 10; Alcohol use disorders test = 35, >12), as well as suffering from a major depressive episode.

In his psychiatric history, he had been admitted to other departments of psychiatry three times for the same reason between 1989 and 2009. Psychiatrists concluded he was suffering from a bipolar disorder coupled with alcohol abuse. His father, whom Mr. B described as a lonely man with many hobbies, had autistic traits and was being treated for bipolar disorder. At the age of 8, he had no school friends and was only interested in reading books. He had no interest in playing with other children or in sports. Although no significant delay was ever reported in language or academic functioning, ever since his childhood, Mr. B. has been described as aloof. He walks on tiptoe, cannot ride a bicycle or tie his shoelaces, and his handwriting is described as “clumsy”. During his early adult life, he excelled academically, studying political science and international relations before obtaining a degree in ethnological science. When he was diagnosed with bipolar disorder in 1989, he stopped studying, and has been employed only for short periods.

His developmental history, as well as his current clinical picture, is consistent with ASD diagnosis according to the DSM-IV criteria and quantitative screening and diagnostic measures, such as the Autism Spectrum Questionnaire (AQ [7]). A comprehensive neuropsychological battery was administered. Mr. B.’s score on the Wechsler Adult Intelligence Scale - 3rd edition (WAIS - III) indicated that he has an average IQ (FSIQ = 92), with higher verbal abilities than visuo-perceptive skills, and a significantly slow processing speed, probably due to dyspraxia and attentional difficulties. In memory tasks, Mr. B. is able to encode and retrieve symbolic verbal information, such as information about his restricted interests (e.g., newspapers, history textbooks), but encoding and retrieval of other types of verbal information are difficult. With most of the visual episodic memory tasks, his results were below average, especially with those that relied more on attentional resources. His social cognition and empathy skills as a whole are impaired. Furthermore, Mr. B.’s neuropsychological profile is characterized by difficulties with set-shifting and cognitive flexibility (in the Trail Making Test, Hayling test, Six Elements Test, and with verbal fluency tasks), weak central coherence—as observed in his copy of the Rey-Osterrieth Complex Figure as well as his highly developed memory for historical and cultural facts (e.g., “Information” subtest of the WAIS-III)—and social cognition impairments. His neuropsychological profile is consistent with both the clinical picture already described (behavioral and social rigidity, restricted interests, and impairments of mental attribution) and the major neuropsychological theories applicable to ASDs.

Mr. B.’s treatment for his mood disorder in 2009 was 2 g valproic acid per day and 200 mg lamotrigine. To avoid delirium tremens, his alcohol withdrawal was managed with 30 mg diazepam per day. He received 900 mg acamprosate three times per day to prevent an alcohol relapse but was unable to stop smoking. After a three-month stay in hospital, he returned home and continued his outpatient psychiatric and social care in our department. Nevertheless, he was regularly hospitalized for alcohol intoxication combined with behavioral disorders and depression. We increased the daily dosage of valproic acid to 2.5 g and lamotrigine to 400 mg, and in 2012 his mood disorder treatment was changed to 2 g valproic acid and 200 mg quetiapine per day.

The patient’s alcohol dependency was first treated unsuccessfully with 50 mg per day naltrexone for more than 3 months. We then switched to baclofen, which was titrated over a 5- week period up to 170 mg per day. However, he was unable to refrain for even 2 h from buying a bottle of beer when he left the hospital. After the HFA diagnosis, his follow-up care was re-organized and adjusted to suit his clinical and cognitive functioning. Currently, his inpatient stays are programmed in advance, last 3 days, and occur once a month. He has psychiatric and psychological outpatient appointments once and twice a week, respectively. They are scheduled on the same days every week to encourage attendance and to limit the anxiety associated with a change in routine. In addition to these environmental modifications, his psychological follow-up was designed according to a cognitive remediation and CBT-inspired framework, the aims being to improve his cognitive flexibility and problem-solving abilities, by means of behavioral exercises involving role-playing and goal management training [8], as well as his social cognition abilities. Emotional awareness and social role-playing were included at later stages of his treatment. Mr. B. is now able to identify and express some of his emotions. Appointments always follow the same pattern, namely, identification of his emotional state, followed by a review of his past and future goals for the day, and discussion of a topical political issue. After a year of twice-weekly treatment, Mr. B.’s social behavior is more reciprocal and flexible. His independent functioning has also improved. He honors his appointments and tends to his apartment; nurses are able to provide home care; and his alcohol consumption is limited to 3 cans of beer per day.

**Mr. T.,** a 55 year-old divorced man, was sent to us for treatment for his addictions in 2011. After consulting many psychiatrists and neurologists over the past few decades, he had recently been diagnosed with Asperger Syndrome by a specialized clinic. With respect to his psychiatric history, he was hospitalized in 1973 for anorexia, and he had suffered from recurrent depressive episodes that started in 2002 and were treated, to no avail, with (successively) sertraline (200 mg per day for 3 months), fluoxetine (40 mg per day for 6 months) and venlafaxine (250 mg per day for 1.5 months). At the end of 2002, a neurologist treated him with 50 mg methylphenidate per day for suspected ADHD, a diagnosis related to difficulties with working memory and attentional tasks identified in a neuropsychological assessment. The diagnosis was confirmed by a psychiatrist and the treatment was continued. As the patient was afraid of becoming dependent on his treatment and highly anxious after taking it, he decided to take 20 mg only when he had to give a lesson. However, his anxiety was so great that he decided to give up treatment.

His brother has been diagnosed with an ASD. With respect to Mr. T’s autobiographical history, he presented difficulties with social skills and had no friends. He felt different from others, and his interests were restricted to languages, including German, Modern Greek, and French. After attending a private primary school, he obtained a master’s degree.

Mr. T. was addicted to strong black tea. He reported drinking 10 cups of tea every morning to improve his concentration and attentional abilities. A few hours after drinking this large quantity of tea, he felt anxious and complained of diarrhea, tremors, and epigastric pain. This anxiety lasted all day. Social contact with Mr. T. was difficult, although he was very talkative. His interactions were one-sided. It required a lot of effort on his part to understand other people’s behavior, and he avoided eye contact. He found some sensory information such as certain smells, like perfume, and noises made by high heels, very distressing. Most of the time, because of these sensory abnormalities, his social difficulties, and the anxiety associated with change, he felt irritable, anxious, and impulsive. With impairments in empathy and social cognition, dysfunction in attention, working memory, and executive abilities, and a detailed, rather than global, information processing style (i.e., weak central coherence), his neuropsychological profile was consistent with his clinical picture and the HFA diagnosis. In the evenings, he was so overwhelmed by his work responsibilities that he drank two bottles of wine before going to sleep. Because alcohol impaired his cognitive and emotion-regulation abilities, Mr. T. found himself trapped in a vicious cycle of dependency on psychostimulants and alcohol. His high tea consumption helped him to improve his attentional and organizational abilities, but in the evening, his high level of excitation and anxiety delayed sleep onset, and Mr. T. found relief in drinking two bottles of wine each night. There was no day of abstinence, and because he found it impossible to give up strong tea and alcohol, he became dependent on them (Alcohol Use Disorders Identification Test = 24).

We persuaded Mr. T. to start by reducing his alcohol consumption. During the withdrawal phase, 20 mg diazepam was prescribed per day, mostly in the evening, together with 900 mg acamprosate 3 times per day. The dose of diazepam was rapidly lowered, but the patient still experienced anxiety in the evening and relapsed. Given that he refused to try an alternative pharmacological treatment, his psychiatric medical care was discontinued, and he started CBT and remediation cognitive therapy, according to the principles described in the case of Mr. B. However, unlike Mr. B., before beginning CBT and executive function remediation sessions, Mr. T. had 12 sessions to try to improve his working memory by means of exercises of increasing difficulty [9]. Furthermore, the aim of Mr. T.’s CBT and executive function remediation was to improve his organizational and initiation skills, which seemed to be a contributing factor to maintaining his high levels of anxiety and some of his addictive behavior. The patient reported some alleviation of executive and working memory difficulties after therapy, but long-term feedback is not available, because Mr. T. is no longer one of our patients.

## Discussion

Our report describes the cases of two adults with HFA who were diagnosed late and who consumed high quantities of alcohol and psychostimulants. A first analysis of these descriptions could lead one to suppose that their SUD was explained by their psychiatric co-morbidities, namely depressive and anxiety disorders, which are often associated with addictive behaviors [10, 11]. Indeed, Mr. B had a mood disorder during the period of treatment. Concerning Mr. T, past depressive episodes were reported but this was no longer the case at the time of our assessments. Furthermore, both patients reported that their anxiety was associated with their behavioral and social rigidity. Given the strong link between anxiety and social rigidity, it is difficult to distinguish which of them is particularly involved in alcohol use in this population. As Matthew Tinsley reports in his book, social skill impairments associated with autism could contribute to alcohol dependency in patients with psychiatric disorders [4]. Godfrey *et al.* [12] showed that a belief that alcohol may be able to enhance social skills and reduce anxiety predicts the amount that alcohol-dependent individuals consume. In our study, Mr. B. and Mr. T. reported that they drank to reduce their anxiety associated with unexpected events, sensory abnormalities, and social events, and to improve their social skills. With regard to schizophrenia, an FMRI study showed that patients who suffered from co-morbid SUD (alcohol or cannabis) had increased activation in the right superior parietal cortex and left medial prefrontal cortex, both regions involved in social cognition, and scored higher on a self-report scale for subjective emotional experience than did non-abusing patients [13]. These results suggest that socio-emotional processing may be less impaired in dual-diagnosis (schizophrenia and SUD) patients. In addition, Mr. B. and Mr. T. presented with attentional and executive dysfunction and attempted to improve their performance with psychostimulants such as nicotine, caffeine, and tea. Kumar *et al.* [14] suggest that cognitive and behavioral impairments in patients with autism could be treated with psychostimulants such as methylphenidate or nicotinic agonistic agents. In fact, both our patients took psychostimulants to improve their neurocognitive abilities, and in both cases, their clinical picture was masked by their SUD, which resulted in a delayed diagnosis of their developmental illness. Acknowledging the clinical and neurocognitive particularities of autism spectrum disorders, which seemed particularly relevant in both our patients for understanding the SUD, gave rise to psychotherapeutic changes that enabled them to improve their overall functioning.

## Conclusion

Teaching addictologists and psychiatrists how better to diagnose adult patients with autism could be useful from two perspectives. First, it could allow for the identification and better treatment of SUD in autism, the prevalence of which may be underestimated. Second, it could also permit the identification of specific autism-related mechanisms, such as cognitive inflexibility and change-related anxiety, which may be a vulnerability trait for developing substance-use disorders. Unless such mechanisms are addressed, it is unlikely these patients will be encouraged to change their SUD.

## Consent

Written informed consent was obtained from the patients for publication of their case reports and any accompanying images. A copy of the written consent is available for review by the Editor of this journal.
